# Stress-Associated and Growth-Dependent Mutagenesis Are Divergently Regulated by c-di-AMP Levels in *Bacillus subtilis*

**DOI:** 10.3390/ijms24010455

**Published:** 2022-12-27

**Authors:** Karen Abundiz-Yañez, Hilda C. Leyva-Sánchez, Eduardo A. Robleto, Mario Pedraza-Reyes

**Affiliations:** 1 Department of Biology, Division of Natural and Exact Sciences, University of Guanajuato, P.O. Box 187, Guanajuato 36050, Gto., Mexico; 2 School of Life Sciences, University of Nevada, Las Vegas, NV 89154, USA

**Keywords:** stress-associated mutagenesis, cyclic-di-AMP, *Bacillus subtilis*

## Abstract

A previous proteomic study uncovered a relationship between nutritional stress and fluctuations in levels of diadenylate cyclases (DACs) and other proteins that regulate DAC activity, degrade, or interact with c-di-AMP, suggesting a possible role of this second messenger in *B. subtilis* stress-associated mutagenesis (SAM). Here, we investigated a possible role of c-di-AMP in SAM and growth-associated mutagenesis (GAM). Our results showed that in growing cells of *B. subtilis* YB955 (*hisC952*, *metB25* and *leuC427*), the DACs CdaA and DisA, which play crucial roles in cell wall homeostasis and chromosomal fidelity, respectively, counteracted spontaneous and Mitomycin-C-induced mutagenesis. However, experiments in which hydrogen peroxide was used to induce mutations showed that single deficiencies in DACs caused opposite effects compared to each other. In contrast, in the stationary-phase, DACs promoted mutations in conditions of nutritional stress. These results tracked with intracellular levels of c-di-AMP, which are significantly lower in *cdaA*- and *disA*-deficient strains. The restoration of DAC-deficient strains with single functional copies of the *cdaA* and/or *disA* returned SAM and GAM levels to those observed in the parental strain. Taken together, these results reveal a role for c-di-AMP in promoting genetic diversity in growth-limiting conditions in *B. subtilis*. Finally, we postulate that this novel function of c-di-AMP can be exerted through proteins that possess binding domains for this second messenger and play roles in DNA repair, ion transport, transcriptional regulation, as well as oxidative stress protection.

## 1. Introduction

Microorganisms continuously face abrupt changes in natural ecosystems that are detrimental to life. However, they rapidly respond to such challenging conditions by activating different strategies of cell survival [1]. These responses are coordinated by signal transduction pathways usually activated by second messengers, resulting in alterations in gene expression and adjustments in cellular physiology [2,3]. A group of second messengers is synthesized from nucleotide precursors, adopting distinctive chemical structures, and are distributed across all domains of life [2,4]. Bacteria can produce different nucleotide-based second messengers, including the classical cyclic-adenosine-monophosphate (c-AMP), the alarmone guanosine-(penta)tetraphosphate [(p)ppGpp] and of most recent interest, cyclic-di-guanosine-monophosphate (c-di-GMP) and cyclic-diadenosine-monophosphate (c-di-AMP) [2,4]. The latter is synthesized by diadenylate cyclases (DACs) from two ATP molecules and hydrolyzed by specific phosphodiesterases [4,5]. All known enzymes that produce c-di-AMP share a catalytically active diadenylate cyclase (DAC) domain which is complemented by a regulatory domain. Based on this modular arrangement, five classes of DACs possessing different types of regulatory domains have been identified in bacteria: namely, DisA, CdaA, CdaS, CdaM, and CdaZ [6,7].

The Gram-positive bacterium *B. subtilis* employs three different DACs (DisA, CdaA and CdaS) to synthesize c-di-AMP [8,9]. The most conserved class of DAC is represented by CdaA [10]. In *B. subtilis*, this protein physically interacts with GlmM, an essential enzyme that catalyzes an early step in peptidoglycan biosynthesis. This interaction likely links cell wall homeostasis to CdaA activity and thus to c-di-AMP levels in the cell [10].

A developmental DNA damage-dependent checkpoint role during the early stages of *B. subtilis* sporulation has been attributed to DisA [11]. It has been shown that DNA lesions diminished both DisA activity and c-di-AMP levels, which results in a sporulation delay [11,12,13]. Most recently, we showed that ROS-promoted DNA lesions activate the checkpoint function of DisA, and together with the transcriptional repair factor Mfd, DisA was found to coordinate excision repair events for efficient *B. subtilis* spore outgrowth [14,15]. Therefore, CdaA and DisA seem to play a role in protecting vegetative *B. subtilis* cells from DNA-damaging agents [16]. Interestingly, CdaS, a spore-specific DAC, does not contribute to DNA repair, but its function is required for spores to return to vegetative growth [17].

During post-exponential growth, *B. subtilis* activates mechanisms that promote genetic variability, which allows this bacterium to escape from growth-limiting conditions. This process has been termed stress-associated mutagenesis (SAM) and takes place in non-dividing bacteria when cells experience a non-lethal selective pressure [18,19]. Previous results from a proteomic analysis demonstrated a relationship between nutritional stress and dysregulation in the levels of DACs and other proteins that degrade or interact with c-di-AMP, suggesting a possible role of this metabolite on *B. subtilis* SAM [20]. In this study we investigated the role of CdaA, DisA and c-di-AMP levels in SAM and growth-associated mutagenesis (GAM). Overall, our results revealed that c-di-AMP produced by DACs CdaA and DisA is needed to prevent mutagenesis during growth; however, in non-dividing or nutrient-limiting conditions, this cyclic dinucleotide may activate programs that produce genetic diversity, which increases the likelihood of escaping growth-limiting conditions.

## 2. Results

### 2.1. DisA and CdaA Are the Main Sources of c-di-AMP in Vegetative Cells of Strain B. subtilis YB955

**Figure 1:**
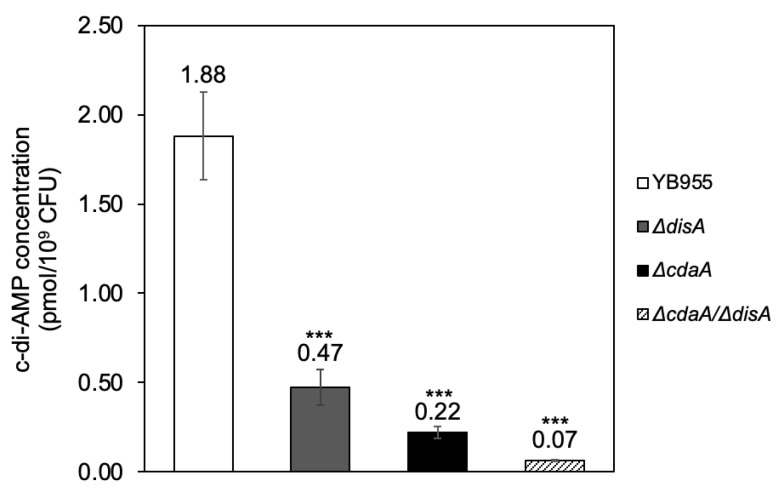
Intracellular c-di-AMP levels of *Bacillus subtilis* YB955 (open bar), Δ*cdaA* (dark grey bar), Δ*disA* (black bar) and Δ*cdaA*/Δ*disA* (hatched bar) were determined in vegetative cells (grown to an OD_600nm_ = 1.0) by competitive ELISA quantification as described in Materials and Methods. Data show the average from three independent determinations ± standard deviations. *** represent significant statistical differences compared to the parental strain, as determined by a one-way analysis of variance (ANOVA) and Tukey Honest Significant Difference (HSD) test (*p* = 2.07 × 10^−8^, *p* = 3.43 × 10^−9^, *p* = 1.28 × 10^−9^, respectively).

DACs DisA and CdaA are implicated in DNA repair in vegetative cells of *B. subtilis* [16]. However, the contribution of these DACs and consequently the c-di-AMP levels produced by these proteins to mutagenic events occurring in vegetative cells from the exponential and stationary phase of growth has remained elusive. To investigate the effects of DACS on mutagenesis, we generated mutant strains of *disA* and/or *cdaA*. To avoid potential polar effects, we designed gene constructs in the integrative plasmid pMUTIN4, which only disrupt *disA* and/or *cdaA* as described in Materials and Methods. The level of c-di-AMP was also determined by competitive ELISA in these strains. Our results revealed that the single disruption of *disA* or *cdaA* decreased the concentration of c-di-AMP by ~75% and ~89%, respectively, compared to the parental strain YB955 (Figure 1).

Notably, vegetative cells of the mutant strain Δ*disA/*Δ*cdaA*, almost completely lost their capability to produce c-di-AMP; this strain displayed a ~96% reduction of the concentration of this second messenger relative to the parental strain proficient for both DACs (Figure 1). Also, the contributions of each DAC to c-di-AMP levels (1.41 and 1.22 pmol/10^9^ cells for DisA and CdaA, respectively) amount to higher values than what is observed in the parental strain and suggest that activity of these enzymes is regulated when both are active. Altogether, these results strongly indicate that CdaA synthesizes more c-di-AMP than DisA and that both DACs are the main sources of this cyclic dinucleotide in vegetative cells of *B. subtilis* YB955.

### 2.2. DisA and CdaA Prevent Spontaneous Mutagenesis in Growing B. subtilis Cells

**Figure 2:**
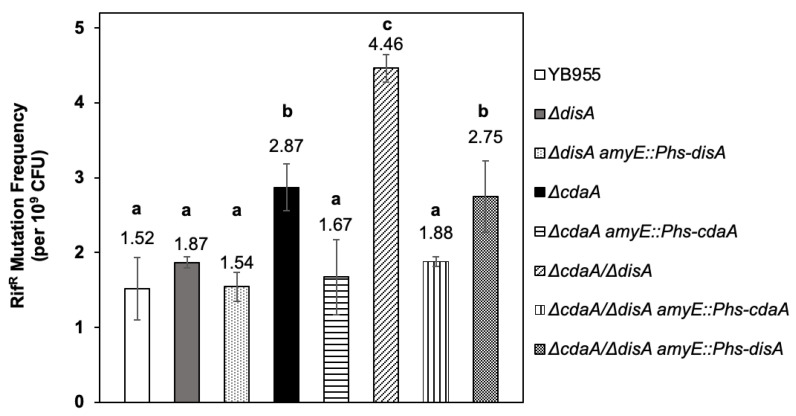
Spontaneous mutation frequencies to rifampicin resistance (Rif^R^) of *Bacillus subtilis* YB955 (open bar), Δ*disA* (dark grey bar), Δ*cdaA* (black bar) and Δ*cdaA*/Δ*disA* (hatched bar) were determined during exponential growth. Complemented strains Δ*disA amyE::*P*hs-disA* (light stippled bar), Δ*cdaA amyE*::P*hs*-*cdaA* (horizontal stripped bar), Δ*cdaA*/Δ*disA amyE::*P*hs-cdaA* (vertical stripped bar), and Δ*cdaA*/Δ*disA amyE::*P*hs-disA* (dark stippled bar) were also tested. Data show the average from three independent experiments, each one in sextuplicate ± standard deviations. All strains were supplemented with IPTG 0.25 mM. Different letters (a, b, c) represent significant statistical differences between means; the same letter denotes no significant difference, as determined by a one-way ANOVA and multiple mean comparison by Tukey HSD test (all *p*-values < 0.001).

After demonstrating that mutant strains Δ*disA*, Δ*cdaA* and Δ*cdaA/*Δ*disA* are decreased in the levels of c-di-AMP, we investigated the contribution of these factors to mutagenic processes taking place on exponentially growing *B. subtilis* cells. To this end, the spontaneous mutation frequency to Rif^R^ was determined for strains with single or double disruptions in *disA* and *cdaA*. The results revealed no significant differences in mutagenesis levels between the *disA* and WT strains; however, the disruption of *cdaA* or both genes did increase ~2 and ~3 times the mutagenesis levels, respectively, compared to the parental YB955 strain (Figure 2).

These mutagenic effects can be attributed to the disruption of *cdaA*, or both *cdaA* and *disA*, as the expression of genes encoding these proteins from the IPTG-inducible P*hs* promoter restored the mutagenic levels to Rif^R^ in the *cdaA* and *cdaA*/*dis*A mutant strains (Figure 2). Altogether, these results suggest that DisA, CdaA and c-di-AMP counteract spontaneous GAM in *B. subtilis*.

### 2.3. CdaA and DisA Exhibit Divergent Responses to H_2_O_2_-Induced Mutagenesis

**Figure 3:**
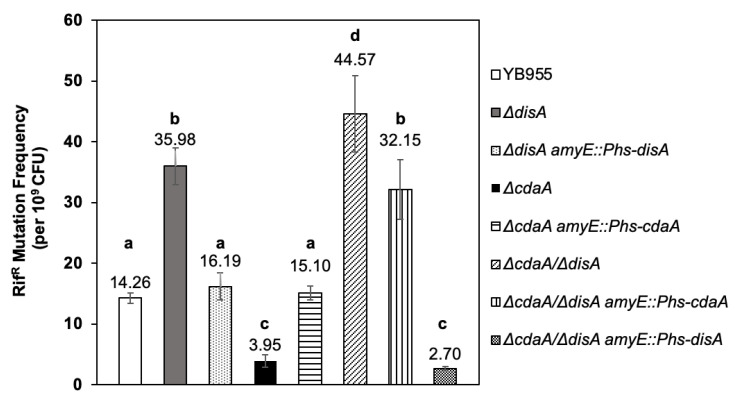
Hydrogen peroxide induced mutation frequencies to rifampicin resistance (Rif^R^) of *Bacillus subtilis* YB955 (open bar), Δ*disA* (dark grey bar), Δ*cdaA* (black bar) and Δ*cdaA*/Δ*disA* (hatched bar) were determined during exponential growth. Complemented strains Δ*disA amyE::*P*hs-disA* (light stippled bar), Δ*cdaA amyE::*P*hs-cdaA* (horizontal striped bar), Δ*cdaA*/Δ*disA amyE::*P*hs-cdaA* (vertical striped bar), and Δ*cdaA*/Δ*disA amyE::*P*hs-disA* (dark stippled bar) were also tested. Data show the average from three independent experiments, each one by sextuplicate ± standard deviations. All strains were supplemented with IPTG 0.25 mM. Different letters (a, b, c, d) represent significant statistical differences between means, the same letter denotes no significant difference, as determined by a one-way ANOVA and multiple mean comparison by Tukey HSD test (all *p*-values < 0.001).

A previous report showed that strains deficient for CdaA but not for DisA are sensitized to hydrogen peroxide treatment [16]. Therefore, we investigated how the disruption of *disA*, *cdaA*, or both, impacted H_2_O_2_-induced Rif^R^ mutagenesis. Interestingly, while the mutagenesis levels promoted by hydrogen peroxide increased ~2.5 times in the *disA* strain, a decrease of ~3.6 times was observed in the *cdaA* strain, compared to the parental strain (Figure 3).

The mutagenesis elicited by H_2_O_2_ increased ~3.1 times in the strain with double disruption in *disA* and *cdaA*, compared to the parental strain YB955 (Figure 3). Furthermore, the reintegration of *disA* or *cdaA* reestablished the mutation frequencies promoted by the oxidizing agent in the strains with single or double disruption in *disA* and *cdaA*, demonstrating that the mutagenesis effects were caused by the disruption of these genes (Figure 3). In conjunction, these results unveil divergent functions in the prevention (DisA) or promotion (CdaA) of mutagenic events elicited by the ROS-promoter agent H_2_O_2_, in exponentially growing *B. subtilis* cells.

### 2.4. CdaA and DisA Are Required to Prevent Mitomycin-C induced DNA Damage

**Figure 4:**
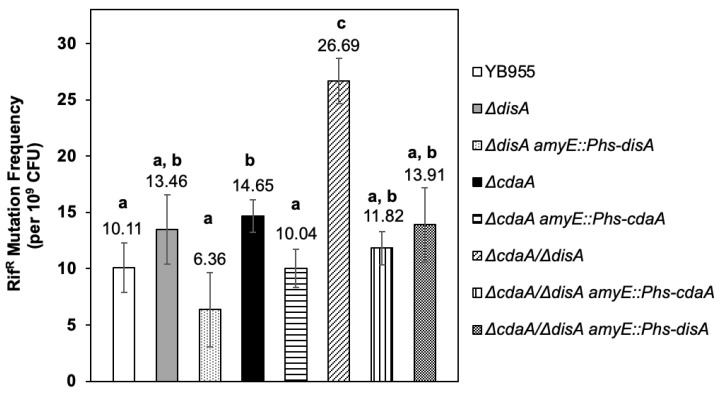
Mytomycin-C induced mutation frequencies to rifampicin resistance (Rif^R^) of *Bacillus subtilis* YB955 (open bar), Δ*disA* (dark grey bar), Δ*cdaA* (black bar) and Δ*cdaA*/Δ*disA* (hatched bar) were determined during exponential growth. Complemented strains Δ*disA amyE::*P*hs-disA* (light stippled bar), Δ*cdaA amyE::*P*hs-cdaA* (horizontal striped bar), Δ*cdaA*/Δ*disA amyE::*P*hs-cdaA* (vertical striped bar) and Δ*cdaA*/Δ*disA amyE::*P*hs-disA* (dark stippled bar), were also tested. Data show the average from three independent experiments, each one by sextuplicate ± standard deviations. All strains were supplemented with IPTG 0.25 mM. Different letters (a, b, c) represent significant statistical differences between means, the same letter denotes no significant difference, two letters in the same bar (a, b) mean statistically equal to both, group a and group b, as determined by a one-way ANOVA and multiple mean comparison by Tukey HSD test (all *p*-values < 0.05).

We next investigated the role of DisA and CdaA in Rif^R^ mutagenesis elicited by the alkylating agent Mitomycin-C (M-C). The results of these assays revealed that, in comparison with the YB955 parental strain, the strains with single disruption in *disA* or *cdaA*, exhibited a slight increase in M-C elicited mutagenesis (Figure 4).

Importantly, the mutant Δ*cdaA/*Δ*disA* exhibited a higher increase (~2.0 times) in the mutagenesis levels promoted by M-C, compared to the parental strain YB955 and single Δ*disA* or Δ*cdaA* strains (Figure 4). Of note, reintegration of *disA* or *cdaA* suppressed the mutagenic effects promoted by M-C in the strains tested, which eliminates the possibility that other linked or co-transcribed genes are influencing this type of mutagenesis (Figure 4). Taken together, these results suggest that DisA and CdaA are needed to counteract GAM elicited by M-C.

### 2.5. DisA and CdaA Are Required for SAM in B. subtilis

**Figure 5:**
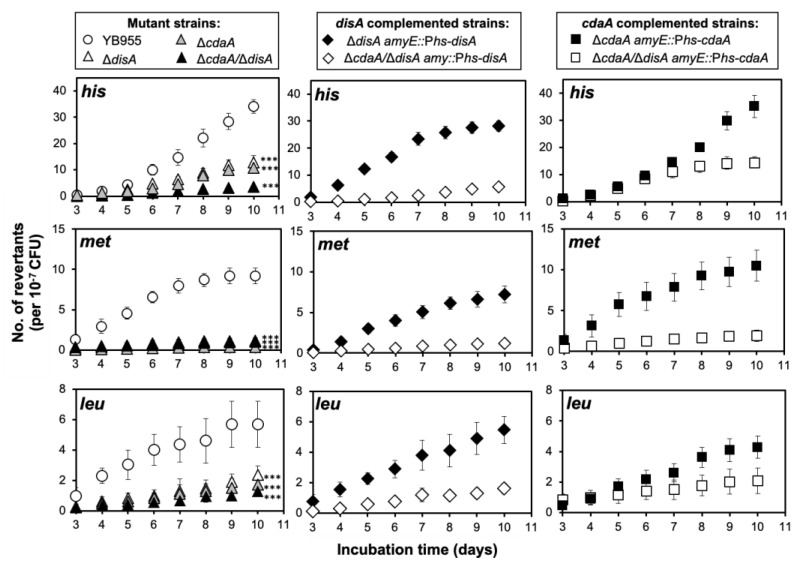
Frequencies of stress-associated reversions for *his, met*, and *leu* mutant genes of *B. subtilis* strains YB955 (open circles), Δ*cdaA* (dark grey triangles), Δ*disA* (open triangles) Δ*cdaA*/Δ*disA* (black triangles) Δ*disA amyE::Phs-disA* (filled diamonds), Δ*cdaA*/Δ*disA amyE::*P*hs-disA* (open diamonds), Δ*cdaA amyE::*P*hs-cdaA* (filled squares) and Δ*cdaA*/Δ*disA amyE::*P*hs-cdaA* (open squares) as described in Materials and Methods. Data represent counts from six plates averaged from three independent experiments normalized to initial cell titters ± standard deviations. All strains were supplemented with IPTG 0.25 mM. *** represent significant statistical differences compared to the parental strain, as determined by a one-way ANOVA and Tukey HSD test (all *p*-values < 0.001).

A previous study reported that *B. subtilis* starved cells displaying an altered response to c-di-AMP and its associated metabolic factors, exhibited an unbalanced pool of dNTPs [20]. This observation prompted us to determine if c-di-AMP influenced SAM. Then, the *B. subtilis* strains, harboring disruptions in *disA* and/or *cdaA* as well as the chromosomal auxotrophies *hisC952*, *metB5* and *leuC427*, were used to determine frequencies of reversion to prototrophy under starving and growth-limiting conditions. Compared to the parental strain YB955, the inactivation of *disA* or *cdaA* led to a decreased production of colonies with a His^+^, Met^+^ or Leu^+^ phenotype; of note, this effect was exacerbated in the strain disrupted for both, *cdaA* and *disA* (Figure 5).

**Figure 6:**
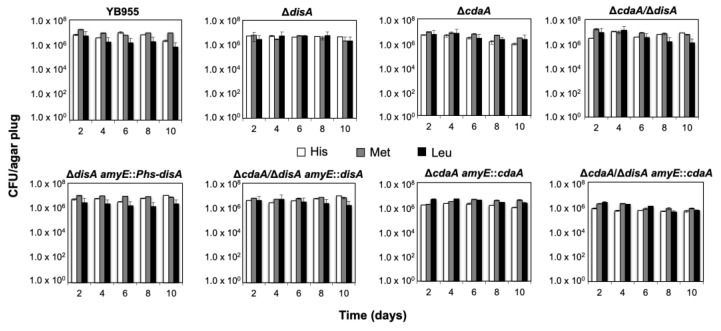
Determination of cell viability throughout SAM reversion assays of *B. subtilis* strains YB955, Δ*cdaA*, Δ*disA* and Δ*cdaA*/Δ*disA*, and of reintegrated strains Δ*cdaA amyE::*P*hs-cdaA*, Δ*cdaA*/Δ*disA amyE::*P*hs-cdaA*, Δ*disA amyE::*P*hs-disA* and Δ*cdaA*/Δ*disA amyE::*P*hs-disA*. Three portions of agar (plugs) were taken from free colonies sites of MM Spizizen plates, every third day, the plugs were extracted and resuspended in 1X SSMM, subjected to serial dilutions and plated on LB media to determine viable counts as described in Materials and Methods. Data represent the average CFU per plug of agar of three determinations ± standard deviations. No significant differences were found.

These effects can be attributed to the single or simultaneous disruption of *disA* and *cdaA*; in support of this contention, the independent expression of these genes from an IPTG-inducible promoter reestablished the *hisC*, *metB*, and *leuC* reversion frequencies to the levels of the strain YB955 (Figure 5). Furthermore, as expected, the production of His^+^, Met^+^ and Leu^+^ prototrophs, in the *disA/cdaA* strain, reached levels such as those exhibited by the strains with single disruptions, following reintegration of *disA* or *cdaA*, respectively (Figure 5). Analyses of survival rates of the strains tested showed that despite small fluctuations, the colony forming units count kept constant, during the ten-day period of the experiments, confirming that the differences observed in the SAM assays were due to the loss of DisA, CdaA, or both proteins and not to variations in cell viability (Figure 6).

### 2.6. Disruption of disA and/or cdaA Promotes the Appearance of Triple His^+^ Met^+^ Leu^+^ Prototrophs

The strain *B. subtilis* YB955, which is auxotrophic to His, Met and Leu due to the chromosomal nonsense *hisC*952 (amber), *met*B5 (ochre) and missense *leuC*427 mutations, has been widely employed to study mutagenesis in amino-acid-starved cells [18,19]. It has been shown that in this strain, a high percentage of the Met^+^ prototrophs obtained, are derived from tRNAs suppressor mutations [18].

To investigate if DACs influences the generation of suppressor mutations, the percentage of suppressor mutations in the parental and mutant *B. subtilis* strains used in this study was determined. Our results revealed that a fraction of the His^+^ colonies obtained of the *cdaA* and *disA*/*cdaA* strains on SAM assays were also Met^+^ prototrophs, suggesting that suppressor mutations on tRNAs gave rise to these revertants (Table 1). This result is in agreement with a previous report for parental strain YB955 in which 20% of His^+^ revertants also exhibited a Met^+^ phenotype and were indeed suppressor mutations [18].

Regarding the *disA* strain, in reference to the parental strain YB955, an increase of ~two times in the percentage of His^+^ colonies with the Met^+^ phenotype was observed, suggesting that disruption of *disA* promotes the formation of ochre tRNA mutations that suppress the His^-^ phenotype. In the case of Met^+^ revertants, in the parental strain YB955 and in all mutant strains, more than 80% of colonies also exhibited a His^+^ phenotype, suggesting that they were generated by ochre tRNA suppressor mutations (Table 1). Of note, these results paralleled those previously reported for the strain YB955 [18]. Interestingly, our suppressor analysis revealed that disruption of *disA*, *cdaA* or both genes promoted the production of colonies exhibiting a triple His^+^ Met^+^ Leu^+^ phenotype, as some of the His^+^ Met^+^ revertants, also grew on SSMM lacking leucine (Table 1). As previously shown [18], none of the Leu^+^ prototrophs produced by the strain YB955 grew in MM selective for His^+^ or Met^+^, strongly suggesting that these revertants resulted from intragenic mutations. Altogether, these results suggest that fluctuations in the c-di-AMP levels elicit a hypermutagenic state that positively impacts the survival of nutritionally stressed *B. subtilis* cells.

## 3. Discussion

Here we investigated the role of DACs and c-di-AMP in the mutagenic processes occurring during growth as well as in stationary-phase cells of *B. subtilis* subjected to amino acid starvation. To this end, we independently or simultaneously disabled *disA* and/or *cdaA* encoding the two DACs that synthesize c-di-AMP in vegetative cells. Overall, the results revealed that c-di-AMP can play divergent roles counteracting GAM but stimulating SAM, thus, maintaining the fidelity of the genome or promoting genetic diversity as affected by the metabolic status of the cell.

As *cdaA* is the first gene of the *cdaA-cdaR-glmM-glmS* operon [9,21], we reasoned that replacement of this gene with an antibiotic cassette could abolish expression of the downstream essential genes *glmM* and *glmS*, which are required for *B. subtilis* cell wall synthesis [21,22]. To avoid polar effects on these genes, we generated a construct in the integrative vector pMUTIN-4 that disrupted *cdaA* through a single cross-over recombination event and allowed expression of the *cdaR-glmM-glmS* cistrons from an IPTG-inducible Ps*pac* promoter [23]. Following this approach, we successfully disrupted *cdaA* in the parental strain YB955 and an isogenic DisA-deficient strain (Figure S1). However, during selection of the transformant colonies and culturing in complex media, both strains required IPTG for a robust growth (Figure S1). Thus, this strategy avoids polar effects on *glmM-glmS* and generated viable strains with disruptions in *cdaA* and/or *disA*, in the genetic background *B. subtilis* YB955, a prophage-cured strain that contains the *hisC952*, *metB5*, and *leuC427* alleles [18]. It must be noted that previous reports showed that *B. subtilis* strains deficient for *disA* and *cdaA* as well as a triple *disA*/*cdaA*/*cdaS* mutant were able to grow in minimal medium with low concentrations of potassium [9,10].

c-di-AMP has been shown to regulate important cellular processes in *B. subtilis*, including potassium and cell wall homeostasis, DNA-damage checkpoint events during sporulation and spore/germination outgrowth and protection against genotoxic compounds [8,9,10,11,12,13,14,15,16,24]. Our results provide evidence for a novel function of this metabolite in modulating bacterial mutagenesis. Notably, the levels of c-di-AMP inversely correlated with the degree of spontaneous Rif^R^ mutagenesis in growing *B. subtilis* cells. Consistently, the *disA* strain, which showed higher levels of this metabolite, produced less mutations than the *cdaA* strain. Furthermore, the disruption of both, *disA* and *cdaA*, which dramatically reduced the production of c-di-AMP, resulted in the highest levels of spontaneous Rif^R^ mutagenesis in growing *B. subtilis* cells. These results suggest that the levels of c-di-AMP may activate cellular processes that modulate spontaneous mutagenic events in growing *B. subtilis* cells.

A previous report showed that CdaA but not DisA, protects vegetative cells of *B. subtilis* from hydrogen peroxide [16], a mutagenic agent that causes oxidative DNA damage and promotes several types of base substitutions [25]. Here we found that DisA counteracted the mutagenesis induced by H_2_O_2_; contrastingly, CdaA promoted this type of mutagenesis. The checkpoint properties of DisA can be activated by genetic insults of oxidative nature or its repair intermediates [14,15]. Accordingly, during *B. subtilis* spore outgrowth, the absence of AP-endonucleases, which are base excision repair components that process ROS-promoted DNA lesions, activates the checkpoint function of DisA and protects the outgrowing cells [14]. This is in agreement with what we showed here; the loss of DisA can negatively impact the ability of *B. subtilis* to withstand the mutagenic effects promoted by H_2_O_2_.

In contrast to DisA, CdaA is a membranal dimeric protein with no predicted functions in DNA repair, which plays critical roles in osmotic regulation and cell wall maintenance [9,10,24]. Therefore, the promutagenic effects induced by H_2_O_2_ in the *cdaA*-deficient strain could be indirect and derived from factors that compromise the homeostasis of the cell envelope. Accordingly, cell envelope stressors, including, antibiotics, heat, ethanol as well as H_2_O_2_ activate the expression of genes under control of the extracytoplasmic function factor σ^M^ (ECM) [8,26]_._ The transcription of *disA* and *radA* is simultaneously induced following activation of the σ^M^ response [27]. Notably, *radA*, whose encoding product has been implicated in repair of DNA damage and recombination, can also physically interact with DisA and counter its DAC activity [27]. This interaction may impact not only the levels of c-di-AMP but also the scanning activity of DisA [12,13] and presumably the processing of ROS-promoted lesions by RadA. Therefore, as previously reported [15], the accumulation of oxidative DNA lesions and/or recombination intermediates can cause replication/transcription conflicts that elicit low-fidelity replication events and that promote Rif^R^ mutagenesis in the *cdaA* strain.

Our results showed that *disA* and *cdaA* play, independently, a minor role in the mutagenic events promoted by M-C; however, the double disruption of these genes significantly increased the mutagenesis levels, above to those exhibited by the strains bearing single disruptions in *disA* or *cdaA* (Figure 4). These results suggest that DisA and CdaA, influence the response to damage inflicted by bifunctional alkylating agents via independent pathways.

Two possible scenarios must be considered to explain these results. Firstly, it has been reported that RuvB, which is part of the LexA regulon [28], antagonizes the DAC and DNA scanning activities of DisA, which eliminate potentially mutagenic lesions from replicating chromosomes [29]. We speculate that, in the absence of DisA, DNA interstrand crosslinks induced by M-C accumulate and activate the SOS-response [30], promoting RecA/RuvB recombination events [31], error-prone replication/repair and mutagenesis [15]. Secondly, the mutagenic effects promoted by M-C can be also attributed to *yqhB*, whose transcription is regulated by the general stress and SOS responses [21,30,32]. YqhB counteracts the noxious effects inflicted by oxidative and electrophilic stresses [21]. Results from bioinformatic analyses indicate that the stress protein YqhB, (i) adopts a dimeric structure and (ii) possesses a CBD domain that interacts with c-di-AMP and activates its oxidoreductase function (Abundiz-Yañez and Pedraza-Reyes, Unpublished Results). Therefore, it is feasible to anticipate a direct impact on GAM in cells deficient in these cellular pathways.

Previous reports suggest that the unfavorable metabolic conditions operating in starved and non-growing bacteria promote SAM [18,19].

Here, we show that fluctuations in the levels of c-di-AMP, via inactivation of *disA* and *cdaA*, impacted SAM and result in a dramatic decline in the reversion frequencies of the mutant alleles *hisC*, *metB* and *leuC* (Figure 5). These effects could be exerted through NrdR a transcriptional repressor of the *nrdEF* operon, which encodes *B. subtilis* ribonucleotide reductase (RNR) [21,33]. A previous report revealed that c-di-AMP can allosterically interact with NrdR and inactivate its repressor activity [24,34]. In starved cells, inactivation of NrdR triggers *nrdEF* transcription, RNR synthesis, and increases in the levels of the dNTP pools, which affects the fidelity of DNA synthesis [33]. In support of these contentions, a recent report, showed that the genetic disruption of *nrdR* and the subsequent derepression of RNR, promoted *B. subtilis* SAM [33].

DarB, another c-di-AMP target, could be implicated in *B. subtilis* SAM. It was shown that the c-di-AMP-DarB complex interacts with RelA to regulate the synthesis of the alarmone (p)ppGpp [35]. In amino acid starved bacteria, this alarmone can activate the stringent response by decreasing the levels of GTP and inactivating the repressor CodY [36,37]. CodY derepression, increases bacterial survival by activating the expression of genes required for the synthesis of methionine, histidine, and arginine, among others amino acids [37,38,39]. In connection with these observations, it has been shown that derepression events mediated by GreA and Mfd influence the reversions frequencies occurring in starved YB955 cells [40]. Notably, low-fidelity repair of ROS-promoted lesions, was presumably implicated in these processes, as the absence of the oxidized guanine (GO) and excision repair systems increased such mutagenic events via low-fidelity DNA polymerases PolX, PolY and YqjW [41,42,43].

These observations, together with experimental evidence presented in this report, support the notion that c-di-AMP functions as a cellular signal that switches on the expression of genetic regulons that promote genetic diversity and allow bacteria to escape from growth-limiting conditions.

## 4. Materials and Methods

### 4.1. Bacterial Strains Plasmids and Growth Conditions

All bacterial strains and plasmids used in this study are listed on Table 2. *B. subtilis* YB955 is a prophage-cured strain that contains the *hisC952*, *metB5* and *leuC427* mutated genes [18] and was routinely propagated in Penassay Broth (PAB) (antibiotic medium 3; Difco Laboratories, Sparks, MD). When required, media was supplemented with erythromycin (Ery, 5 µg/mL), spectinomycin (Spc, 100 µg/mL), chloramphenicol (Cm, 5 µg/mL), rifampicin (Rif, 10 µg/mL) or isopropyl-β-D-thiogalactopyranoside (IPTG, 0.25 mM). *E. coli* was cultured in Lysogeny-Broth (LB) supplemented if required with 100 μg of ampicillin/mL. Liquid cultures were incubated at 37 °C in a shaker adjusted to 250 rpm and monitored with a colorimeter set at 590 nm. For stationary phase mutagenesis experiments and determination of suppressor mutations, Spizizen Salts Minimal Medium (SSMM) was used. SMMM was composed in % (*w*/*v*) by: 1X Spizizen Salts (SS) [(NH_4_)_2_SO_4_ 0.2%; K_2_HPO_4_ 1.4%; KH_2_PO_4_ 0.5%; sodium citrate 0.1% and MgSO_4_.7H_2_O 0.02%] and glucose 0.5%, supplemented with Isoleucine and Glutamic Acid 50 µg/mL, 0. 2X of Ho-Le trace elements [MgCl_2_•6H_2_O 12.5%, CaCl_2_ 0.55%, FeCl_2_•6H_2_O 1.35%, MnCl_2_•4H_2_O 0.1%, ZnCl_2_ 0.17%, CuCl_2_•2H_2_O 0.043%, CoCl_2_•6H_2_O 0.06% y Na_2_MoO_4_•7H_2_O 0.02%], 200 ng/mL of the selection amino acid (histidine, methionine, or leucine) and 50 µg/mL of the remaining two amino acids [18].

### 4.2. Construction of Mutant Strains

To obtain a null mutant in the *cdaA* gene of *B. subtilis*, a 317-bp internal fragment, encompassing from nucleotides +91 to +407, of the gene ORF, was amplified with the specific nucleotide primers 5′-CGAAGCTTGTGATACGCGGCACGAAAGC-3′ (forward) and 5′-GCGGATCCCGCTCAATGGTCAGCAGGGC-3′ (reverse) (restriction sites HindIII and BamHI, respectively, underlined) and high-fidelity Vent DNA polymerase (New England BioLabs; Ipswich, MA, USA). This fragment was cloned into the pJET1.2/blunt vector (Thermo Scientific, Waltham, MA, USA). Subsequently, the *cdaA* fragment was ligated into the HindIII/BamHI sites of the vector pMUTIN-4-cat [41] and the resulting construct pPERM1663 (Table 2) was transformed and replicated in *E. coli* DH5α using standard techniques [44]. To obtain a genetic construct for disrupting *disA*, an internal 309-bp internal fragment, encompassing nucleotide positions +274 to +583, of the gene ORF, was amplified with the specific nucleotide primers 5′-CGGAATCCCGAATACTCAGCTGATGC-3′ (forward) and 5′-CCGGATCCGACAGACAAGACATCACTG-3′ (reverse) (restriction sites EcoRI and BamHI, respectively, underlined) and was cloned in the integrative vector pMUTIN-4 following the strategy for disrupting *cdaA* above described resulting in construct pPERM732 [14].

To obtain the single *disA* or *cdaA* mutants, competent cells of strain *B. subtilis* YB955, prepared according to the protocol of Boylan et al. 1972 [45] were, respectively, transformed with the constructs pPERM1663 and pPERM732 (Table 2). These procedures generated *B. subtilis* strains with disruptions in *disA* (PERM1647; Ery^r^) and *cdaA* (PERM1687; Cm^r^), respectively (Table 2). Transformants were selected in solid media supplemented with IPTG and Ery or Cm, as needed. The strain with simultaneous deficiencies in *cdaA* and *disA* (PERM1665) was obtained as follow. Competent cells of *B. subtilis* PERM1647 (*disA*::Ery) were transformed with plasmid pPERM1663 (Table 2). Transformants were selected in medium supplemented with IPTG and amended with Ery and Cm. For corroboration of the correct insertion of the constructs into the genes of interest, chromosomal DNA was extracted from *B. subtilis* transformants and PCR was performed using forward and reverse specific oligonucleotide primers aligning on the disrupted *cdaA* gene or the *lacZ* gene contained in the pMUTIN-4 vector, respectively. The PCR product was analyzed by agarose gel electrophoresis performed by standard techniques [44] (Figure S1).

### 4.3. Construction of Complementing Strains

We generated a construct to overexpress *cdaA*, through amplification of the complete ORF (819-bp) of *cdaA* plus 72-bp downstream of its stop codon by PCR using chromosomal DNA from *B. subtilis* YB955, Vent DNA polymerase, and specific forward (5′-GCGCATGCATGGCTTTTGAGGATATCCC-3′) and reverse (5′-GCGTCGACACCGCCACATAAAGCAAG-3′) oligonucleotide primers that contained SphI and SalI restriction sites (underlined), respectively. The PCR product was first cloned into the pJET1.2/blunt vector and subsequently ligated into the SphI/SalI sites of the integrative vector pDR111 (Table 2). The resulting construct (pPERM1845; Table 2) was linearized and used to transform competent cells of strains *B. subtilis* PERM1687 (*cdaA*::*cat*) and *B. subtilis* PERM1665 (*cdaA*::*cat*; *disA*::*ery*).

A construct to overexpress d*isA* was generated as follows. The full ORF of *disA* (1080-bp) and 200-bp downstream of the stop codon was first amplified by PCR using chromosomal DNA from *B. subtilis* YB955, Vent DNA polymerase and specific forward (5′-GCAGGTCGACATGGAAAAAGAGAAAAAACGG-3′) and reverse (5′-GCAGGCTAGCGTACAAACAATTCAGGTATCA-3’) oligonucleotide primers, bearing SalI and NheI restriction sites (underlined), respectively. After cloning into pJET1.2/blunt, the SalI/NheI PCR fragment was ligated into pDR111 previously digested with the same enzymes to generate the construct pPERM1728 (Table 2). This plasmid was linearized and transformed into competent cells of strains *B. subtilis* PERM1647 (*disA*::*ery*) and *B. subtilis* PERM1665 (*cdaA*::*cat*; *disA*::*ery*). Chromosomal DNA isolated from transformants colonies selected in medium with the appropriate antibiotics and specific oligonucleotide primers forward (5′-TTGAGCTCAATGGGGAAGAGAACCGC-3′) and reverse (5′-GATCAAAAGCGGAACCATTCTTC-3′), were used to determine by PCR, the correct insertion of the construct pPERM1845 (P*hs-cdaA*) and pERM1728 (P*hs-disA*) into the *amyE* locus.

### 4.4. Immunological Quantitation of c-di-AMP

To quantify the concentration of c-di-AMP in the parental strain YB955 and mutants lacking *cdaA* and/or *disA*, a competitive ELISA kit (Cayman Chemical Company; Ann Arbor, MI, USA) was used. For preparation of cell lysates, the parental and mutant strains were independently grown to an OD_600nm_ of 1.0; at this point, samples (2 mL) of each culture were collected and the cells were pelleted by centrifugation (4800× *g*/3 min/4 °C). The pellets were washed twice with 20 mM Tris/HCl buffer pH = 7.5 and stored at −20 °C until used. Next, cells resuspended in 0.3 mL of 20 mM Tris/HCl buffer pH = 7.5 were incubated with 0.1 mL of lysozyme (10 mg/mL) and 0.002 mL of DNAse I (1 U/µL) for 15 min at 37 °C. After disruption, cell lysates were centrifuged for 10 min at 4800× *g*/4 °C and the supernatants were employed to prepare half serial dilutions, samples (0.05 mL) of these dilutions were transferred in triplicate to the wells of an enzyme-linked immunosorbent assay (ELISA) plate pre-coated with a goat anti-mouse IgG along with different amounts of a standard c-di-AMP solution to obtain a standard curve. 0.05 mL of a c-di-AMP tracer linked to horseradish peroxidase (HRP) and 0.05 mL of a mouse anti-c-di-AMP monoclonal antibody were added to each well. Plates were incubated for 2 h, washed and then supplemented with 0.0175 mL of the colorimetric substrate 3,3′,5.5′-tetramethylbenzidiene (TMB) to determine the concentrations of the bound c-di-AMP-HRP tracer. Plates were incubated with TMB for 30 min, then the reaction was stopped with 0.075 mL of HRP-stop solution and plates were read in a microplate reader (Varioskan Flash Multimode Reader; Thermo Scientific, Pittsburgh, PA, USA) set at 450 nm and quantitation of c-di-AMP was calculated from the standard curve. Results were collected from at least two different dilutions for each sample, per triplicate and adjusted to a specific number of cells determined by viable counts from the cultures of each strain.

### 4.5. Analysis of Mutation Frequencies to Rif^R^

Mutation frequencies to Rif^R^, in the absence or presence of H_2_O_2_ and M-C, were determined as previously described [40]. Briefly, the strains of interest were propagated in PAB medium in a shaker adjusted to 37 °C and 250 rpm to an OD_600nm_ of 0.5; at this point, the culture was split into two subcultures. One of the subcultures was left untreated to determine spontaneous mutagenesis and the other two were supplemented with a lethal dose 25 (LD_25_) of H_2_O_2_ or M-C. The cultures were incubated at 37 °C with constant shaking for an additional period of 12 h. Subsequently, the cells were harvested by centrifugation at 4800× *g* for 10 min at room temperature, washed with 10 mL of 1X SS and resuspended in a final volume of 1 mL of this solution. Finally, aliquots of 0.1 mL were plated in six plates of LB medium supplemented with Rif (10 μg/mL) and incubated at 37 °C; the colonies with a Rif^R^ phenotype were counted after 24 h. The number of cells in the bacterial culture was determined by serial dilution and viable count on LB medium plates. The mutation frequency was reported, for each experiment, as the average number of Rif^R^ colonies per 10^9^ viable cells. These experiments were repeated at least three times for each strain.

### 4.6. Stationary-Phase Mutagenesis Assays

The strains of interest were propagated in sterile flasks containing PAB medium at 37 °C in a shaker adjusted to 250 rpm until 90 min after T_0_ (namely, the time when the slopes of exponential growth and stationary phase intersected). The stationary-phase mutagenesis assays were performed as previously described [18] by plating cell aliquots (100 μL) on six plates of solid Spizizen minimal medium. The concentration of the amino acid used depended on the reversion that was being selected. For instance, to select for His^+^ revertants, 50 µg/mL of methionine and leucine and 200 ng/mL of histidine were added to the medium. Isoleucine and glutamic acid were added as described previously [18] to protect the viability of the cells. When required, the selection medium was supplemented with IPTG (0.25 mM final concentration). The number of revertants from the six plates was scored daily. The initial number of bacteria for each experiment was determined by serial dilution of the bacterial cultures and then by plating the cells on a minimal medium containing all three essential amino acids. The experiments were performed at least three times. To assess cell survival during the period of the stationary phase mutagenesis experiments, we implemented a previously described protocol [16]. Briefly, using a sterile Pasteur pipette, 3 portions of agar (plugs) were taken from free colonies sites of MM Spizizen plates, every third day. Agar plugs were resuspended in 1 mL of 1X SS, serial dilutions were performed and 100 μL aliquots were plated in LB medium from 10^−3^ to 10^−5^ dilutions. The plates were incubated at 37 °C and colonies were counted after 12 h of incubation. These assays were performed in triplicate throughout SAM experiments.

### 4.7. Determination of Suppressor Mutations Generated during Stationary Phase

The percentage of suppressor mutations generated during stationary phase was determined by collecting up to 100 His^+^, Met^+^ and Leu^+^ revertant colonies from the tested strains, generated on days 6 to 8 after plating on the selective SSMM. These colonies were propagated in the corresponding SSMM selective media and subsequently screened for growth on SSMM plates lacking one other amino acid. For example, to determine the percentage of His^+^ suppressor mutations, colonies that were selected on histidine drop-out media were screened for growth on methionine drop-out media. Then, the number of colonies that were Met^+^ were counted and divided by the total number of Hist^+^ colonies that were screened [18].

### 4.8. Statistical Analyses

Statistical analyses to compare the results of the mutants and complemented strains with the parental strain YB955 were performed using R software. A Shapiro–Wilk normality test was performed for all data, as well as homoscedasticity of variances test; after confirming the normality and homoscedasticity of the data, a one-factor analysis of variance (ANOVA) was performed, and means were compared with a Tukey’s honest significance test. The statistical significance used in all tests was *p* = 0.05.

## Figures and Tables

**Table 1:** Growth of stationary-phase DAC-deficient revertants on alternative selective media ^a^.

Revertants	Number of Revertants That Grew/Number Tested (% of Revertants That Grew) on a Minimal Medium Lacking:			Revertants with His^+^ Met^+^ Leu^+^ Phenotype (% of Total)
	His	Met	Leu	
**YB955**				
His^+^	104/104 (100)	15/104 (14.4)	0/104 (0)	0 (0)
Met^+^	68/78 (87.2)	78/78 (100)	0/78 (0)	0 (0)
Leu^+^	0/113 (0)	0/113 (0)	113/113 (100)	0 (0)
**Δ*cdaA***				
His^+^	110/110 (100)	15/110 (13.6)	1/110 (0.9)	1 (0.9)
Met^+^	75/76 (98.7)	76/76 (100)	5/76 (6.6)	5 (6.6)
Leu^+^	4/115 (3.5)	9/115 (7.8)	115/115 (100)	3 (2.6)
**Δ*disA***				
His^+^	168/168 (100)	47/168 (27.9)	2/168 (1.2)	0 (0)
Met^+^	71/84 (84.5)	84/84 (100)	14/84 (16.7)	14 (16.7)
Leu^+^	4/159 (2.5)	0/159 (0)	159/159 (100)	0 (0)
**Δ*cdaA/*Δ*disA***				
His^+^	96/96 (100)	18/96 (18.7)	5/96 (5.2)	1 (1.0)
Met^+^	63/73 (86.3)	73/73 (100)	2/73 (2.7)	2 (2.7)
Leu^+^	1/102 (1.0)	1/102 (1.0)	102/102 (100)	1 (1.0)

^a^ His, Met and Leu revertant colonies from days 4, 5 and 6 were tested on 1× SSMM missing one required amino acid (His, Met, or Leu) to screen for suppressor mutations. Plates were scored after 48 h.

**Table 2:** *Bacillus subtilis* strains and plasmids used in this study.

Strain	Genotype or Description ^a^	Reference or Source
YB955	*hisC952 metB5 leuC427 xin-1 Sp*β*^SENS^*	[18]
PERM1647	YB955 *disA::ery*. Em^R^	This study
PERM1665	YB955 *cdaA::cat disA::ery*. Ery^R^ Cm^R^	PERM1647 → PERM1663 ^b^
PERM1687	YB955 *cdaA::cat*. Cm^R^	This study
PERM1729	YB955 *disA*::*ery amyE::Phs-disA*. Ery^R^ Sp^R^	PERM1647 → PERM1728
PERM1730	YB955 *cdaA::cat disA*::*ery amyE:: Phs-disA*. Ery^R^ Cm^R^ Sp^R^	PERM1665 → PERM1728
PERM1853	YB955 *cdaA::cat amyE::Phs-cdaA*. Cm^R^ Sp^R^	PERM1687 → PERM1845
PERM1854	YB955 *cdaA::cat disA::ery amyE::Phs-cdaA*. Cm^R^ Ery^R^ Sp^R^	PERM1665 → PERM1845
**Plasmids**		
pMUTIN-4	Integrative vector for *B. subtilis*. Disrupts the gene after insertion and creates a fusion of the cloned fragment with the *lacZ* gene. Ery^R^	[23]
pMUTIN-4-cat	Integrative vector for *B. subtilis*. Disrupts the gene after insertion and creates a fusion of the cloned fragment with the *lacZ* gene. Cm^R^	[41]
pDR111	pHyperspank integrative vector, Amp^R^ Sp^R^	David Rudner Laboratory
PERM732	pMUTIN-4 plasmid containing an internal fragment of the *B. subtilis disA* gene. Amp^R^	This study
PERM1663	pMUTIN-4-cat plus an internal fragment of the *B. subtilis cdaA* gene. Cm^R^	This study
PERM1845	pHyperspank-*cdaA* Integrative vector with *cdaA* under control of an IPTG inducible P*hs* promoter, Amp^R^ Sp^R^	This study
PERM1728	pHyperspank-*cdaA* Integrative vector with *disA* under control of an IPTG inducible P*hs* promoter, Amp^R^ Sp^R^	This study

^a^ Amp, ampicillin; Cm, chloramphenicol; Ery, erythromycin; Sp, spectinomycin; ^b^ Chromosomal DNA from the strain to left of the arrow was used to transform the strain to the right of the arrow.

## Data Availability

Not applicable.

## Supplementary Materials

The supporting information can be downloaded at https://www.mdpi.com/article/10.3390/ijms24010455/s1.
