# Supplementary material for: Stress-Associated and Growth-Dependent Mutagenesis Are Divergently Regulated by c-di-AMP Levels in Bacillus subtilis
Source: Int J Mol Sci. 2022 Dec 27;24(1):455. doi: 10.3390/ijms24010455 (PMC9820435; doi:10.3390/ijms24010455)
Supplement: Supplementary file 1 [file ijms-24-00455-s001.zip › ijms-1998559-supplementary.pdf]

SUPPLEMENTARY MATERIAL

Stress-associated and Growth-Dependent Mutagenesis are Divergently Regulated by c-di-AMP Levels in *Bacillus subtilis*

Karen Abundiz-Yañez[1], Hilda C. Leyva-Sánchez[2], Eduardo A. Robleto[2], Mario Pedraza-Reyes[1*].

[1]Department of Biology, Division of Natural and Exact Sciences, University of Guanajuato, Guanajuato, México; [2]School of Life Sciences, University of Nevada, Las Vegas, Nevada, USA.

*Address correspondence to Mario Pedraza-Reyes, pedrama@ugto.mx

SUPPLEMENTAL FIGURE S1.

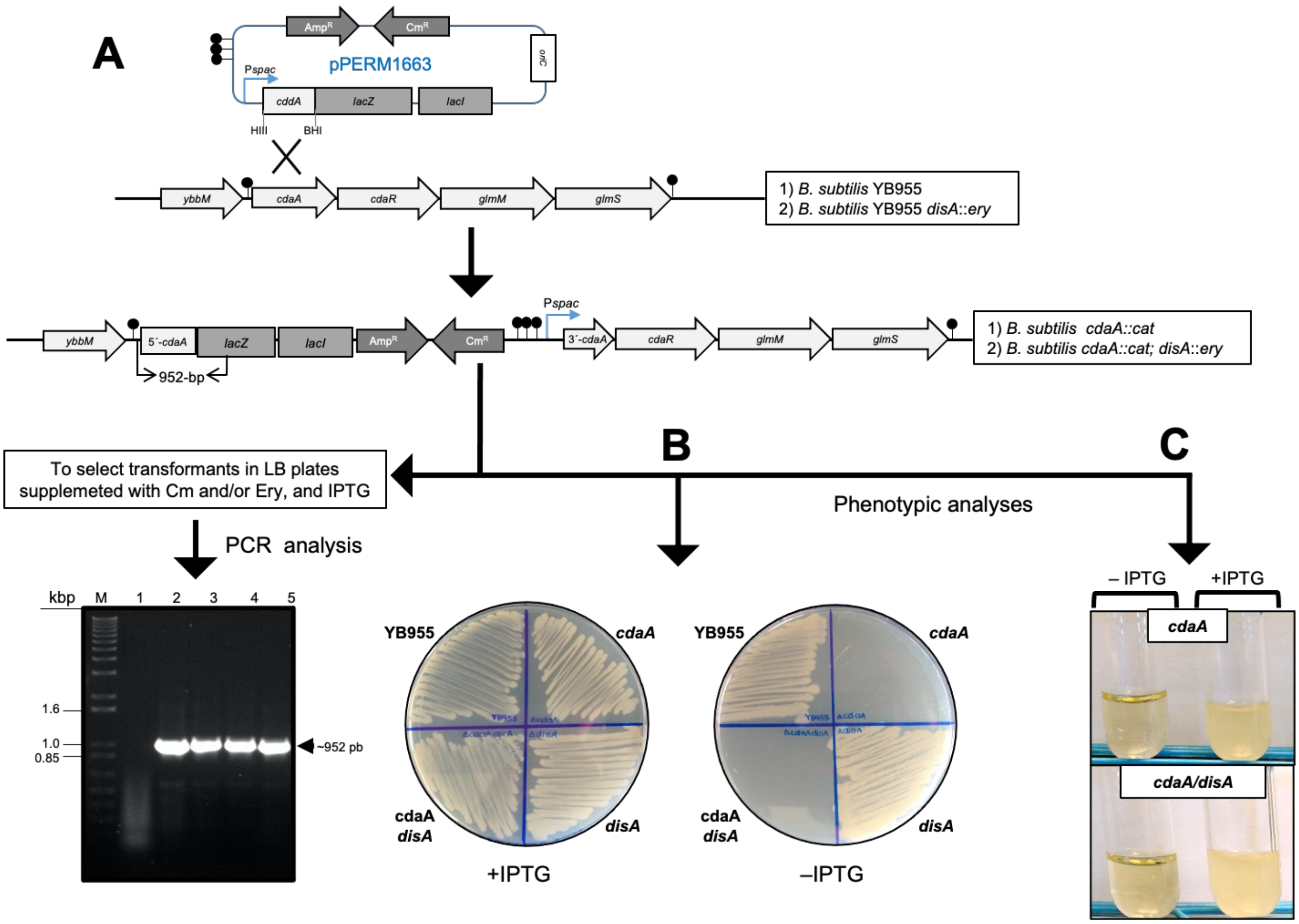

Figure S1. Construction and characterization of *B. subtilis* strains carrying *cdaA* or *cdaA/disA* disruptions. **A)**. The integrative plasmid pPERM1663 (see Materials and Methods) was used to transform competent cells of *B. subtilis* strains YB955 (Parental) and the isogenic derivative strain PERM1647 (*disA*::*ery*), respectively. The Campbell-type recombination event leading to inactivation of *cdaA* in these genetic backgrounds was confirmed by amplification of a 952-bp PCR product employing specific specific oligonucleotide primers as indicated in Materials and Methods. The PCR products from 2 transformant colonies of the *cdaA*::*cat* (Lanes 2, 3) and *cdaA*::*cat*/*disA*::*ery* (Lanes 4, 5) strains were separated in an agarose gel that was stained with ethidium bromide and shown at the bottom left. Lane 1 corresponds to a PCR reaction performed with chromosomal DNA of the parental strain YB955. **B,C)**. Strains deficient for *cdaA* and/or *disA*, as well as the parental strain YB955, were streaked in LB agar plates and incubated for 24 h at 37°C (**B**), or propagated in liquid LB medium for 16 h at 37°C with aeration (250 rpm) (**C**). In both types of cultures, media was supplemented or not with IPTG (0.25 mM), as indicated.
